# Successful Treatment With Intrathecal and Intravenous Polymyxin B-Based Combination Against MDR *Acinetobacter baumannii* Meningitis in Pediatric Patient: A Case Report

**DOI:** 10.3389/fped.2021.564991

**Published:** 2021-07-27

**Authors:** Haiyan Xing, Caiyi Cheng, Yihua Zhang, Yongqing Cai, Xianfeng Wang, Dongmei Deng, Lunshan Xu, Minhui Xu, Jianhong Chen

**Affiliations:** ^1^Department of Pharmacy, Daping Hospital, Army Medical University, Chongqing, China; ^2^Department of Neurosurgery, Daping Hospital, Army Medical University, Chongqing, China

**Keywords:** meningitis, multidrug resistance, *Acinetobacter baumannii*, Polymyxin B, tigecycline, intrathecal administration

## Abstract

**Background:** Nosocomial meningitis with multidrug-resistant (MDR) or extensively drug-resistant (XDR) *Acinetobacter baumannii* is a life-threatening complication in neurosurgery. Treatment of these infections is challenging because of poor penetration of the available antibiotics into the cerebrospinal fluid (CSF). Intrathecal (ITH) or intraventricular (IVT) administration of antibiotics is increasingly used as the last treatment option against MDR/XDR Gram-negative bacteria meningitis not responding to intravenous (IV) regimens. However, pertinent data in pediatric patients is scarce.

**Case Presentation:** A 14-year-old male patient developed meningitis from an MDR strain of *A. baumannii* following endoscopic endonasal resection of craniopharyngioma. Despite a combination therapy involving IV tigecycline, we observed clinical and bacteriologic failure. The patient was then successfully treated with an ITH and IV polymyxin B-based combination. Quantification of tigecycline and polymyxin B in CSF was performed with two-dimensional high-performance liquid chromatography (2D-HPLC) and HDLC coupled with tandem mass spectrometry (HPLC-MS/MS), respectively. Adverse drug reactions (neurotoxicity and skin hyperpigmentation), probably induced by polymyxin B, were acceptable and reversible.

**Conclusions:** The case illustrates ITH and IV Polymyxin B-based combination is an optimal therapeutic option against MDR *A. baumannii* meningitis in this pediatric patient. In the future, real-time PK/PD data obtained from patients during ITH/IVT polymyxin B therapy should be required to optimize polymyxin use with maximal efficacy and minimal adverse effects.

## Introduction

Postoperative meningitis due to multidrug-resistant (MDR) or extensively drug-resistant (XDR) Gram-negative bacteria is a life-threatening complication in neurosurgery. *Acinetobacter baumannii* has emerged as one of the major organisms isolated from the cerebrospinal fluid (CSF) cultures (1). Mortality of nosocomial meningitis caused by *A. baumannii* was reported as high as 72.7% (2). MDR/XDR *A. baumannii* strains possess an impressive armamentarium of resistance mechanisms, resulting in resistance to all, or almost all, commercially available antibiotics (3). This fact has led to the resurrection of polymyxins (polymyxin B and colistin) as salvage therapy (4). Considering the potentially suboptimal pharmacokinetic (PK)/pharmacodynamic (PD) and the increasing prevalence of multidrug resistance, combination therapy is recommended for polymyxins even though there is a lack of solid clinical evidence (5–7). Furthermore, due to the poor central nervous system (CNS) penetration of antibiotics (8), intrathecal (ITH) or intraventricular (IVT) routes have been considered as the last resort for the treatment of ventriculitis/meningitis caused by MDR/XDR Gram-negative bacteria not responding to intravenous (IV) regimens (9). However, the clinical efficacy and tolerability profile of ITH/IVT polymyxins therapy, especially in pediatric patients, remains obscure.

This report presents a case of a 14-year-old male patient who developed meningitis from an MDR strain of *A. baumannii* following endoscopic endonasal resection of craniopharyngioma. The patient has been successfully treated with ITH and IV Polymyxin B-based combination after a failed combined therapy involving IV tigecycline. In addition, concentrations of tigecycline and polymyxin B in CSF were retrospectively determined by two-dimensional high-performance liquid chromatography (2D-HPLC) and HPLC coupled with tandem mass spectrometry (HPLC-MS/MS), respectively (Supplementary Material).

## Case Presentation

**Figure 1:**
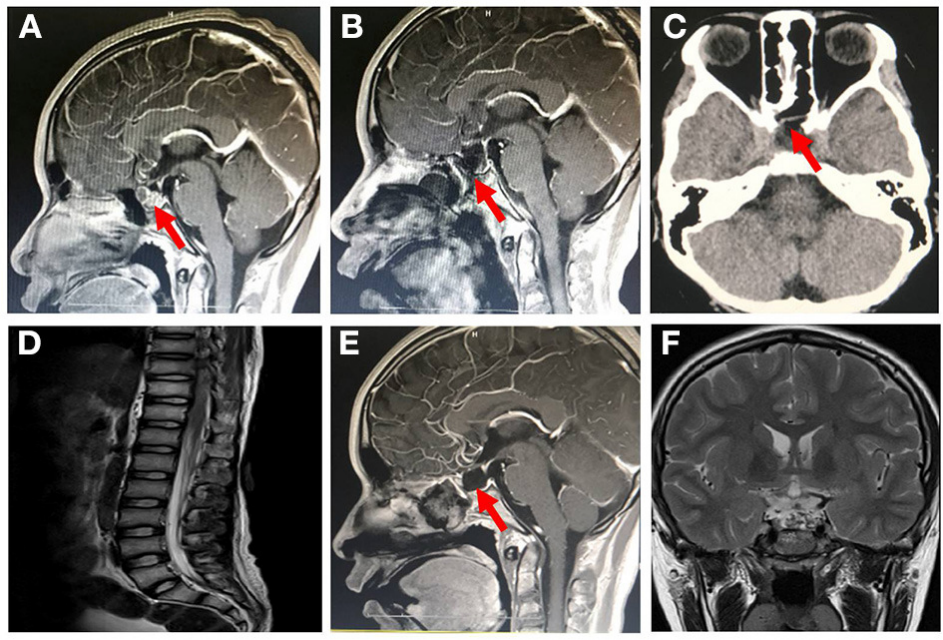
Brain magnetic resonance imaging (MRI) and computed tomography (CT) image after patient admission. **(A)** Sagittal enhanced MRI of pituitary showed enlarged sella turcica with a mass of about 1.2 cm × 1.6 cm × 1.4 cm in it, pituitary stalk extension to the right side and optic chiasm compression pituitary (January 25, 2019). **(B)** Sagittal enhanced MRI of pituitary showed that the craniopharyngioma was completely removed, and the skull base was incompletely covered by mucosal flap (January 29, 2019). **(C)** On day 47 of admission (March 12, 2019), nonenhanced skull CT scan revealed that the cerebrospinal fluid leakage was successfully repaired owing to the skull base reconstruction with mucosal flap. **(D)** Whole-spine MRI revealed no obvious adhesion in the spinal canal after 4 days of polymyxin B treatment (March 19, 2019). Follow-up pituitary MRI revealed **(E)** no recurrence of tumor, well healed mucosal flap in skull base, and **(F)** no obvious hydrocephalus (January 16, 2020).

The patient was admitted to our hospital on January 24, 2019, with blurred vision in both eyes for 1 week. Neurological examinations and laboratory evaluations revealed no abnormality. The patient was diagnosed with craniopharyngioma by neuroradiologic examinations (Figure 1A). Complete tumor removal was achieved *via* the endoscopic endonasal transsphenoidal approach on day 4. Perioperative IV antibiotic prophylaxis with ceftriaxone (2 g) was administered. The intraoperative course of the patient was uneventful. However, the postoperative MRI examination showed an incomplete skull base reconstruction on the following day (Figure 1B).

**Figure 2:**
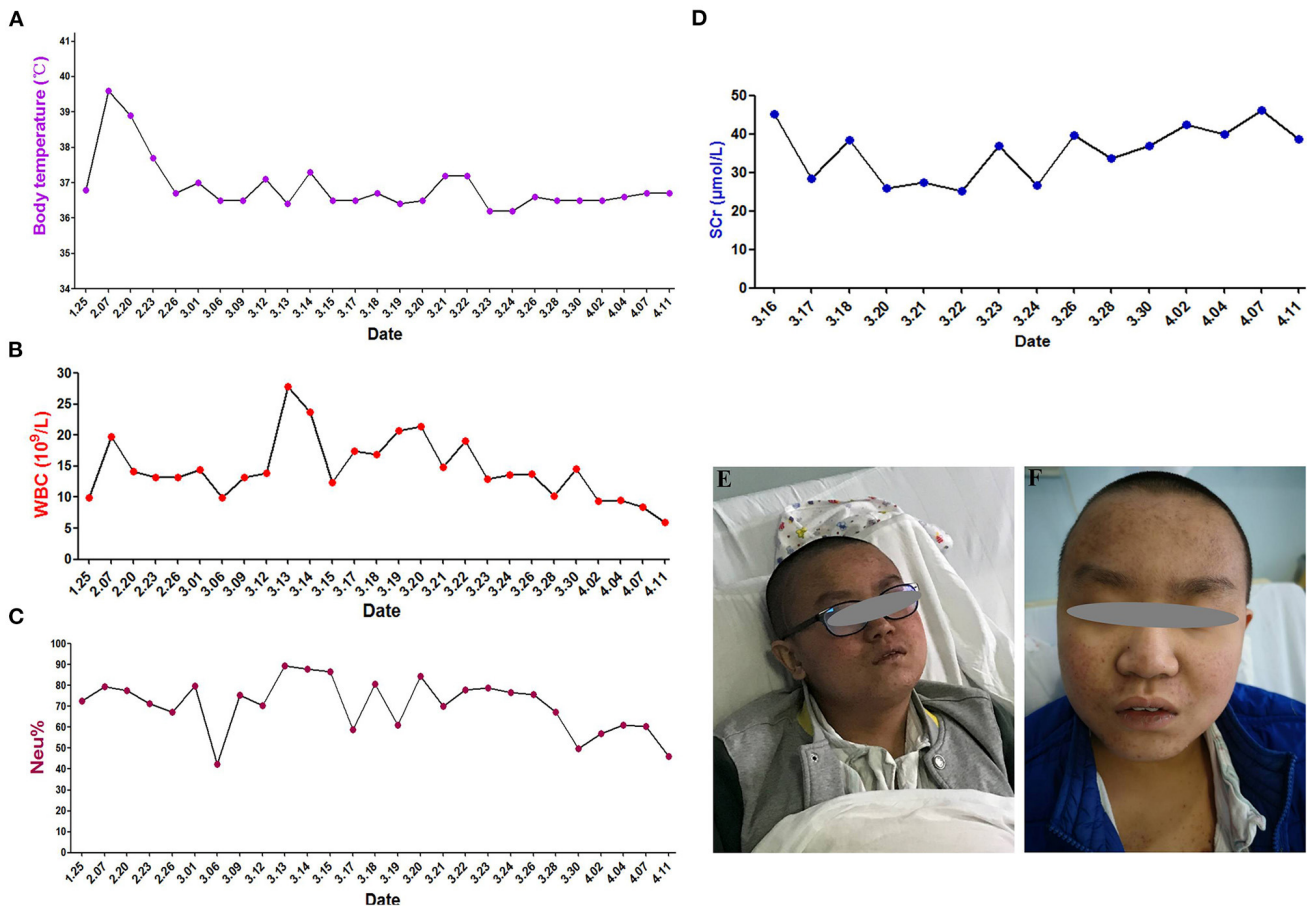
Clinical course and skin hyperpigmentation of the patient. **(A)** Body temperature over the course of treatment. **(B)** White blood cell (WBC) count in the peripheral blood. **(C)** Neutrophil percentage (NEUT%) in the peripheral blood. **(D)** Serum creatinine (SCr) levels. **(E)** Skin hyperpigmentation and dark red papule appeared in the upper part of trunk of patient on the fourth day of polymyxin B treatment (March 18, 2019). **(F)** Skin hyperpigmentation gradually disappeared (January 16, 2020).

**Figure 3:**
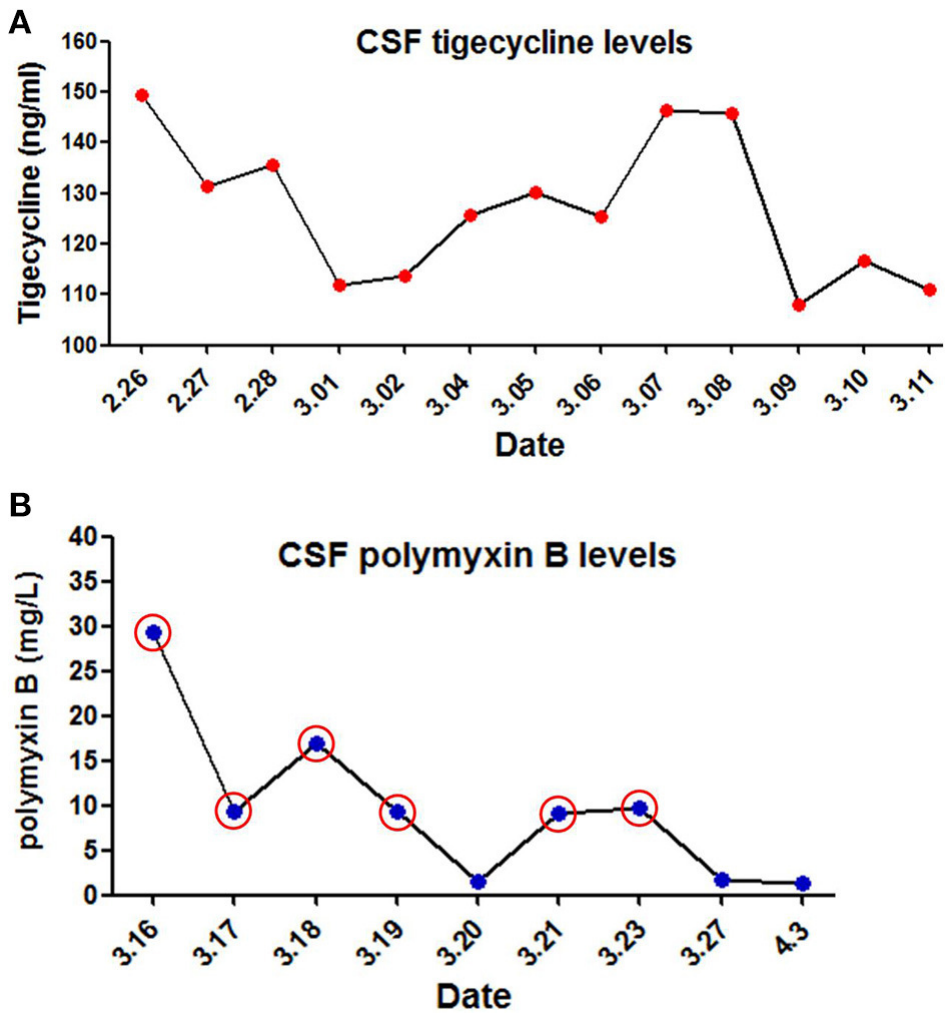
Levels of tigecycline and polymyxin B in cerebrospinal fuid (CSF) of the patient. **(A)** CSF tigecycline concentrations were measured with Two-Dimensional High-Performance Liquid Chromatography (2D-HPLC) method. **(B)** CSF polymyxin B levels were determined using high performance liquid chromatography coupled with tandem mass spectrometry (LC-MS/MS). Red circle represents the day of ITH polymyxin B treatment.

The immediate postoperative recovery of the patient was complicated by an episode of CSF rhinorrhoea. Ten days after surgery (day 14), the patient presented with remittent fever (peak at 39.6°C, Figure 2A) and irritative cough. CSF rhinorrhoea was highly suspected. The patient thus underwent two transnasal endoscopic repairs (on day 14 and 31), in which fibrin glue, dural substitutes, autologous fat tissue graft, and temporary lumbar drainage were used. The laboratory tests for CSF showed a slightly high level of white blood cell (WBC) and total cell counts (Table 1). In contrast, WBC count and neutrophilic granulocyte percentage (NEUT%) in his peripheral blood were significantly higher (Figures 2B,C). Consequently, regardless of negative CSF culture, nosocomial meningitis was considered. IV linezolid (600 mg twice daily) and IV ceftriaxone (2 g twice daily) were initiated empirically (day 15). During follow-up, the patient's fever gradually subsided. However, a sudden onset of fever (38.9°C) occurred on February 20th (day 27), with vomiting and neck rigidity. CSF analysis revealed a WBC count of 0.098 × 10^9^/L, a total protein of 2.41 g/L, and glucose of 2.32 mmol/L (Table 1). The simultaneous blood glucose level was 6.34 mmol/L. CSF culture was positive for MDR *A. baumannii* (Table 2), which was sensitive to tigecycline, amikacin, and minocycline, and intermediate to cefoperazone/sulbactam based on the breakpoints from the Clinical and Laboratory Standards Institute (CLSI) (10). For tigecycline, the U.S. Food Drug Administration (FDA)-approved breakpoints were applied (11). Empiric treatment was quickly replaced by the therapeutic regimen of IV tigecycline (100 mg/day in two doses after a loading dose of 100 mg) combined with meropenem (2 g as a 3-h prolonged infusion every 8 h) and amikacin (7.5 mg/kg twice daily). CSF samples were collected from lumbar drainage before the initiation of the next dose of tigecycline and stored at −80°C for uniform testing. Concentrations of tigecycline were determined by 2D-HPLC with ultraviolet detection, and the signal was recorded at 340 nm (Figure 3A). Though the CSF cultures remained positive for *A. baumannii*, this therapy led to subsequent clinical and laboratory improvement. However, on day 46 after admission, the patient suffered from intolerable diarrhea without noticeable relief after symptomatic treatment. Considering the commonly reported side effects (12, 13), IV therapy with both tigecycline and amikacin was stopped (19 days from the start of IV tigecycline), while the IV meropenem was continued. Meanwhile, IV cefoperazone/sulbactam (3 g every 6 h) and minocycline (orally 200 mg/day in two doses after a loading dose of 200 mg) were added. However, during treatment, gradually decreased CSF drainage was observed. Therefore, the lumbar drainage was removed, while a new sterile lumbar catheter was positioned on day 4 of this therapy. Simultaneously, CSF obtained from the new lumbar drainage was cloudy with a marked increase in protein (3.65 g/L) and WBC count (4.054 × 10^9^/L; Table 1), and the microbiological testing was consistently positive. Fortunately, a nonenhanced skull CT scan revealed successful repair of CSF leak (Figure 1C).

**Table 1:** Laboratory tests for cerebrospinal fuid over the course of treatment.

**Date**	**Cell count (×10^**9**^/L)**	**WBC (×10^**9**^/L)**	**Glucose (mmoL/L)**	**Total protein (g/L)**	**Chlorine (mmoL/L)**
2019.02.07 (D14)	0.040	0.019	3.30	0.27	119.0
2019.02.20 (D27)	2.075	0.098	2.32	2.41	112.4
2019.02.23 (D30)	0.405	0.242	2.86	0.72	122.9
2019.02.26 (D33)	0.643	0.187	1.95	0.79	120.7
2019.03.01 (D36)	0.018	0.015	1.88	0.65	122.0
2019.03.03 (D38)	0.045	0.040	2.95	0.28	125.0
2019.03.06 (D41)	0.020	0.017	3.13	018	122.3
2019.03.09 (D44)	0.009	0.004	2.90	0.16	121.9
2019.03.11 (D46)	0.008	0.002	4.40	0.14	120.9
2019.03.14 (D49)	4.142	4.054	0.33	3.65	118.5
2019.03.15 (D50)	3.649	3.045	0.36	2.23	114.6
2019.03.16 (D51)	0.382	0.371	2.46	1.22	115.7
2019.03.17 (D52)	0.567	0.541	1.38	0.89	112.9
2019.03.18 (D53)	0.150	0.126	2.32	0.94	110.6
2019.03.19 (D54)	1.305	1.255	1.10	1.08	119.2
2019.03.20 (D55)	0.008	0.003	3.26	0.76	97.1
2019.03.22 (D57)	0.412	0.398	2.20	0.56	115.1
2019.03.26 (D61)	0.010	0.007	2.68	0.61	121.1
2019.04.03 (D69)	0.070	0.054	2.90	0.85	123.2
2019.04.11 (D77)	0.050	0.045	3.02	0.74	121.8
**Normal reference ranges**	**0-0.008**	**0-0.008**	**2.5-4.5**	**0.15-0.45**	**120-132**

**Table 2:** Antibiotics susceptibility tests for *Acinetobacter baumannii* in CSF.

**Antibiotics**	**Susceptibility**	**K-B (mm)**	**MIC (μg/mL)**
Meropenem	Resistant	8	N/A
Minocycline	Susceptible	16	N/A
Cefoperazone/sulbactam	Intermediate	18	N/A
Piperacillin	Resistant	6	N/A
Amikacin	Susceptible	22	N/A
Tigecycline	Susceptible	N/A	0.5
Ampicillin	Resistant	N/A	≥32
Cefazolin	Resistant	N/A	≥64
Ceftriaxone	Resistant	N/A	≥64
Cefepime	Resistant	N/A	≥64
Ceftazidime	Resistant	N/A	≥64
Imipenem	Resistant	N/A	≥16
Piperacillin/tazobactam	Resistant	N/A	≥128
Gentamicin	Intermediate	N/A	8
Tobramycin	Susceptible	N/A	≤ 1
Ciprofloxacin	Resistant	N/A	≥4
Levofloxacin	Resistant	N/A	≥8
Trimethoprim-sulfamethoxazole	Resistant	N/A	8

The antimicrobial therapy was changed to ITH polymyxin B (50,000 U once daily for 4 days, then 50,000 U once every other day), IV polymyxin B (450,000 U twice daily), and cefoperazone/sulbactam (3 g every 6 h) on March 15, 2019. Polymyxin B was dissolved in saline up to a total volume of 1 mL and slowly injected into the CSF (14). After each injection, the lumbar drainage was temporarily closed for 60 min to prevent untimely washout of the drug (1). CSF samples for laboratory examinations and concentration determinations were collected from the lumbar catheter before the next dose of polymyxin B. Direct quantification of polymyxin B in CSF was performed with HPLC-MS/MS equipped with electrospray (ESI) ionization interface (Figure 3B). CSF sterilization (a negative CSF culture) was achieved the next day, following remarkable improvement in CSF findings. Unfortunately, hyperpigmentation and dark red papule appeared in the upper part of the patient's trunk, especially on the face and neck on the fourth day of polymyxin B treatment, and gradually aggravated (Figure 2E). Over the next few days, the patient experienced mild memory loss, hypotonia, lumbosacral pain, and sore legs. However, whole-spine MRI revealed no apparent abnormality (Figure 1D), and renal function tests showed normal serum creatinine levels (Figure 2D). Because of the known neurotoxicity and skin hyperpigmentation induced by polymyxin B (15, 16), ITH polymyxin B was replaced with minocycline oral administration on March 24, 2019 (9 days from the onset of ITH polymyxin B), while IV polymyxin B and cefoperazone/sulbactam were continued. Ten days later, IV polymyxin B was discontinued. The signs of meningitis disappeared in a few days. Meanwhile, laboratory tests of CSF and radiological examination returned to normal. Thus, the patient was discharged on April 12, 2019.

At the 12-months follow-up, the patient was in good clinical condition without signs of intracranial infection. Neurologically, vision in both his eyes was normal. Skin hyperpigmentation (Figure 2F) and strength in his limbs were gradually recovered, and the patient could walk normally. Radiological signs were also normal (Figures 1E,F).

## Discussion

This paper reports on a 14-year-old male patient who developed MDR *A. baumannii* meningitis following neurosurgical surgery and subsequent CSF rhinorrhoea. The risk factors such as CSF leakage, lumbar drainage placement, and repeated transnasal endoscopic repair may be significantly associated with subsequent *A. baumannii* meningitis of this patient (17). Nosocomial meningitis due to MDR *A. baumannii* seriously threatens patient survival. However, its treatment is challenging because of limited available therapeutic options, especially in pediatric patients (3).

The frequent emergence of carbapenem resistance of *A. baumannii* has led to the revival in polymyxins and tigecycline as the last resort antimicrobials (18). Since polymyxins were not always available and the susceptibility test for polymyxin B and colistin were not routinely performed in our hospital, tigecycline was initially administered intravenously with meropenem and amikacin according to the *in vitro* susceptibility testing. Although tigecycline is not approved for use in children by FDA, the results of a few available data suggest that it may be a valauble consideration for life-threatening infections in pediatric patients, alone or in combination with other antibiotics, when other therapies are not suitable (19, 20). Moreover, there are some reported cases of postoperative meningitis that were successfully treated with tigecycline (the same dosage regimen as in the present case) as a last line therapy (21, 22). Hence, the combination of IV tigecycline with amikacin and prolonged infusion of meropenem may be a potential alternative for salvage treatment in this case.

Unfortunately, the tigecycline-based combination therapy failed to achieve microbiological eradication despite promising clinical and laboratory improvement. A previous study of tigecycline concluded that the administration of a dose of 1–2 mg/kg q12h in children aged 12 years or older, to a maximum dose of 50 mg, provided PK values similar to those reported to be effective in adults (23). However, our retrospective CSF determination showed that the steady-state concentration of tigecycline was 108.08–149.54 ng/mL, a value far below the FDA breakpoint (24). It suggests that personalized dosing of IV administration of tigecycline is necessary for a patient with MDR *A. baumannii* meningitis to avoid suboptimal drug exposure in the CSF. Thus, further pharmacological studies are required to describe the PK/PD profile of tigecycline in children of different ages and infection types to optimize the dosage regimen. Additionally, as described in recent studies, ITH/IVT administration of tigecycline may be another option for treating bacterial meningitis caused by MDR *A. baumannii* (14, 25). Regarding the safety profile, previous studies showed that the adverse events associated with tigecycline in children include nausea, vomiting, diarrhea, neutrophil engraftment delay, and acute pancreatitis (23). The patient in this case suffered from intolerable diarrhea leading to the discontinuation of tigecycline combination therapy. The result might also be attributed to other factors, such as a worse clinical condition and concomitant drugs (26). Therefore, additional data from controlled clinical studies are required to assess the safety of tigecycline, and particular caution should be given to off-label use of tigecycline, especially in pediatric patients.

Polymyxins have been revived as a last-line defense against difficult-to-treat MDR *A. baumannii* in this pediatric patient following the failure of IV tigecycline. Although limited polymyxin B information is available, especially in pediatric patients, recently available data indicates that polymyxin B has significant PK/PD advantages in contrast to colistin, and its dosage regimens should not be adjusted according to the patient's renal function (27). As IV polymyxins demonstrate poor CNS penetration, ITH and IV routes have been employed to achieve high polymyxin concentrations in the CNS (28, 29). It is reported that the combination of IV and ITH/IVT polymyxins treatment has been used as an effective and safe therapeutic option against CNS infections caused by MDR Gram-negative bacteria, including in critically ill patients and children (30). Furthermore, polymyxin B monotherapy is not an ideal clinical option since dose escalation to achieve sufficiently high concentrations carries risks of rapid-onset nephrotoxicity (31). Thus ITH and IV polymyxin B-based combination was considered salvage therapy for this pediatric patient with lumbar drainage.

To the best of our knowledge the daily dose of polymyxins ranges between 5,000 IU (in infants) and 120,000 IU (divided into two doses) in children (32). The duration of therapy is quite variable (2–4 weeks), and shorter treatments (<1 week) may correlate with higher mortality (28). In this pediatric patient, the combination including a low dosage of ITH polymyxin B was administered. CSF sterilization was rapidly achieved without significant nephrotoxicity. However, the mild neurotoxicity and skin hyperpigmentation caused by polymyxin B was highly suspected, although it was difficult to isolate adverse reactions and clinical outcomes solely from this drug. As reported previously, the onset of polymyxin B-induced neurotoxicity and hyperpigmentation usually occurs shortly after IV infusion or within the first few days of polymyxin therapy and is generally reversible with drug discontinuation (33, 34). The major risk factors associated with neurotoxicity are the extended exposure (duration and concentration) to polymyxins; the presence of medical conditions such as myasthenia gravis, renal impairment, and hypoxia; concomitant use of other medications (e.g., sedatives, anesthetics, narcotics, and muscle relaxants); and gender (33). These findings are in accordance with the suspected adverse reactions observed in the present case. Moreover, recent case reports of polymyxin B-induced skin hyperpigmentation is associated with its position 6 D-Phe, because no such reports have been published on colistin to date (35). It has been reported that penetration of polymyxins into CSF is a crucial indicator for optimizing *in vivo* efficacy and minimizing toxicity (28). However, the PK information of polymyxins in CSF after ITH/IVT delivery remains largely unknown. Our retrospective CSF determination showed a wide range of steady-state concentrations of polymyxin B (1.33–29.41 mg/L), which may be closely associated with rapid bacterial eradication and highly suspected adverse reactions.

The present case enriched the PK information of polymyxin B in CSF, which may improve the clinical practice of polymyxins, especially in pediatric patients. However, it has limitations; for example, only one case has been reported. Additionally, the therapeutic drug monitoring (TDM) of tigecycline and polymyxin B in CSF were retrospectively performed after the end of treatment without TDM-based dosing optimization. In the future, real-time PK/PD data obtained from patients during ITH/IVT polymyxin B therapy should be required to optimize polymyxin use with maximal efficacy and minimal adverse effects.

## Conclusions

This case highlights the issues involved in treating life-threatening *A. baumannii* meningitis of pediatric patients. IV tigecycline should not be recommended for CNS infections of MDR Gram-negative pathogens because of the poor penetration into CSF. ITH administration of polymyxin B is a convenient route, especially in pediatric patients with lumbar drainage. Additionally, combined treatment with IV and ITH polymyxin B is vital for CSF sterilization. However, the efficacy and safety of tigecycline and polymyxins for pediatric CNS infections need to be clarified. Given the narrow therapeutic windows of polymyxin B, it is necessary to identify a practical PK/PD profile for optimizing dosage regimens of ITH/IVT polymyxin therapy.

## Data Availability Statement

The original contributions presented in the study are included in the article/Supplementary Material, further inquiries can be directed to the corresponding author/s.

## Ethics Statement

The studies involving human participants were reviewed and approved by the ethics committee of Daping Hospital. Written informed consent to participate in this study was provided by the participants' legal guardian/next of kin. Written informed consent was obtained from the individual(s), and minor(s)' legal guardian/next of kin, for the publication of any potentially identifiable images or data included in this article.

## Author Contributions

HX: conceptualization. CC: methodology. YZ: case presentation. YC: writing-original draft. XW: data analysis. DD: data collection. LX and MX: validation, writing-review, and editing. JC: supervision and project administration. All authors have read the last version of this manuscript and agree this submission.

## Conflict of Interest

The authors declare that the research was conducted in the absence of any commercial or financial relationships that could be construed as a potential conflict of interest.

## Publisher's Note

All claims expressed in this article are solely those of the authors and do not necessarily represent those of their affiliated organizations, or those of the publisher, the editors and the reviewers. Any product that may be evaluated in this article, or claim that may be made by its manufacturer, is not guaranteed or endorsed by the publisher.
