# Supplementary material for: Successful Treatment With Intrathecal and Intravenous Polymyxin B-Based Combination Against MDR Acinetobacter baumannii Meningitis in Pediatric Patient: A Case Report
Source: Front Pediatr. 2021 Jul 27;9:564991. doi: 10.3389/fped.2021.564991 (PMC8353103; doi:10.3389/fped.2021.564991)
Supplement: Supplementary file 1 [file Data_Sheet_1.docx]

Supplementary Material

# Supplementary Data

Tigecycline levels in cerebrospinal fluid (CSF) were performed with two-dimensional high-performance liquid chromatography (2D-HPLC). The first-dimension column was an Aston SNX5 phenyl chromatographic column (50 mm×4.6 mm, 5 μm). The mobile phase was a 45:55 (V/V) solution of ammonium phosphate (pH adjusted to 7.5 by ammonium hydroxide)-methanol, with a flow rate of 1.2 ml/min. The second-dimension chromatographic column was Aston SC5 C18 (275 mm×4.6 mm, 5 μm). The mobile phase was a 30:50:20 (V/V/V) solution of ammonium phosphate (pH adjusted to 7.4 by ammonium hydroxide)-ammonium phosphate (pH adjusted to 3.0 by ammonium hydroxide)- acetonitrile, with a flow rate of 1.0 ml/min. The detection wavelength was 340 nm.

CSF colistin levels were determined by high performance liquid chromatography coupled with tandem mass spectrometry (HPLC-MS/MS) according to the method as reported by He et al with slight modification. In brief, cerebrospinal fluid samples were precipitated with 5% trichloroacetic acid and then separated on a Shim-pack GIST C18 column (Shimadzu, Kyoto, Japan) using gradient elution at a flow rate of 0.4 mL/min. The selected reaction monitoring transitions were performed at m/z 402.10→101.15 for polymyxin B1, m/z 397.40→101.15 for polymyxin B2 and m/z 578.30→101.15 for polymyxin E2 (Internal standard).

He, J., Gao, S., Hu, M., Chow, D.S., and Tam, V.H. (2013). A validated ultra-performance liquid chromatography-tandem mass spectrometry method for the quantification of polymyxin B in mouse serum and epithelial lining fluid: application to pharmacokinetic studies. *J Antimicrob Chemother* 68**,** 1104-1110.
